# Advanced preparation of plan-view specimens on a MEMS chip for *in situ* TEM heating experiments

**DOI:** 10.1557/s43577-021-00255-5

**Published:** 2022-03-07

**Authors:** Alexey Minenkov, Natalija Šantić, Tia Truglas, Johannes Aberl, Lada Vukušić, Moritz Brehm, Heiko Groiss

**Affiliations:** 1 Christian Doppler Laboratory for Nanoscale Phase Transformations, Center for Surface and Nanoanalytics, Johannes Kepler University Linz, Altenberger Straße 69, 4040 Linz, Austria; 2 Tietz Video and Image Processing Systems GmbH, Eremitenweg 1, 82131 Gauting, Germany; 3 Institute of Semiconductor and Solid-State Physics, Johannes Kepler University Linz, Altenberger Straße 69, 4040 Linz, Austria

**Keywords:** *In situ* TEM, Plan-view, Wedge polishing, Focused ion beam, SiGe

## Abstract

*In situ* transmission electron microscopy (TEM) is a powerful tool for advanced material characterization. It allows real-time observation of structural evolution at the atomic level while applying different stimuli such as heat. However, the validity of analysis strongly depends on the quality of the specimen, which has to be prepared by thinning the bulk material to electron transparency while maintaining the pristine properties. To address this challenge, a novel method of TEM samples preparation in plan-view geometry was elaborated based on the combination of the wedge polishing technique and an enhanced focused ion beam (FIB) workflow. It involves primary mechanical thinning of a broad sample area from the backside followed by FIB-assisted installation on the MEMS-based sample carrier. The complete step-by-step guide is provided, and the method’s concept is discussed in detail making it easy to follow and adapt for diverse equipment. The presented approach opens the world of *in situ* TEM heating experiments for a vast variety of fragile materials. The principle and significant advantage of the proposed method are demonstrated by new insights into the stability and thermal-induced strain relaxation of Ge Stranski–Krastanov islands on Si during *in situ* TEM heating.

**Graphical abstract:**

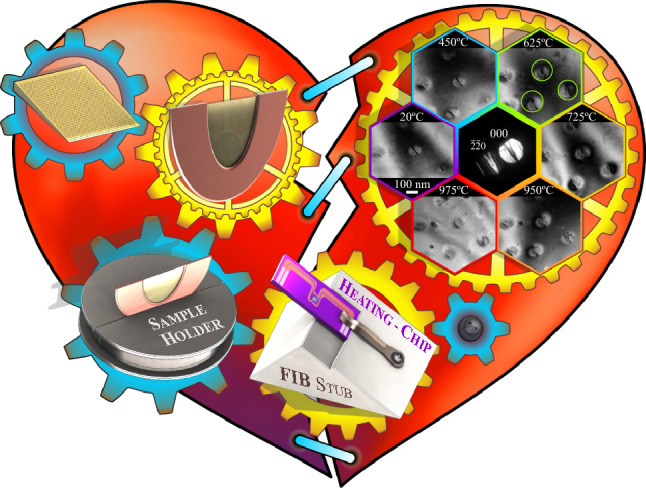

**Supplementary Information:**

The online version contains supplementary material available at 10.1557/s43577-021-00255-5.

## Impact Statement

The quality of the specimens is imperative for *in situ* TEM investigation, which allows real-time observation of structural evolution at the atomic level applying different stimuli. The challenging preparation of a suitable specimen by thinning the bulk material to electron transparency is even more complicated for samples where a free surface must be prepared as a plan-view specimen. However, this kind of sample is vital for gathering detailed three-dimensional information on the material’s structure. The presented method of plan-view samples preparation opens the world of *in situ* TEM heating experiments for a vast variety of fragile materials ranging from MBE-grown semiconductor nanolayers to industrial coatings. Its conceptual advancement is demonstrated by fundamental findings, which can be barely achieved without applying the elaborated method. The synergy of the wedge polishing technique and the advanced FIB workflow allows us to combine the advantages of both approaches minimizing invasive effects such as mechanical load and ion beam illumination. We have also provided a glimpse into the state of the art of various sample preparation techniques, which makes the article suitable not only for experts but also for readers, who would like to make the first steps toward fruitful TEM research.

## Introduction

The development and synthesis of novel materials must be guided and supported by a thorough structural and compositional analysis. In this regard, transmission electron microscopy (TEM) is an excellent tool for in-depth material characterization and can provide synergistic morphological, compositional, and crystallographic information. *In situ* TEM experiments are even more powerful and allow the observation of structural changes while applying different stimuli such as heat. With real-time tracking of the material response, a direct correlation of structural evolution at the nanoscale with transition temperatures is possible and can provide a breakthrough in understanding phase transition mechanisms and the kinetics involved.^1–5^ *In situ* microscopy has gained in the last years even more importance by the introduction of cutting-edge micro-electro-mechanical system (MEMS) chip-based heating holders^6,7^ replacing the conventional holders fitted with macroscopic heating elements for self-supporting specimens.

In order to study material properties in a TEM, the specimen must be electron transparent at the chosen acceleration voltage. For instance, a thickness of 200 nm for Si-based specimens is suitable for selected area electron diffraction (SAED) and energy-dispersive x-ray spectroscopy (EDXS) mapping at around 200 kV. However, for high-resolution scanning TEM (HR-STEM), the specimen thickness has to be reduced to less than 50 nm. It is imperative that the applied sample preparation technique must preserve the original material structure.^8–10^ Thus, the TEM sample production itself constitutes a significant field of study, aiming for the development of novel and improved techniques.

For thin films, two different viewing geometries are of importance. Cross-section geometry assumes that the layer of interest is parallel to the electron beam allowing to investigate thickness changes and intermixing,^4^ or the interfaces at the atomic level, etc., while in plan-view geometry, the electron beam is perpendicular to the surface,^11^ that is, for instance, crucial to obtain information about the morphological evolution of thin layers,^4,12^ grain sizes and orientations^13^ or particle size distributions caused by the de-wetting of continuous films.^14–19^

Self-supporting TEM samples can be produced in cross-section or in plan-view orientation by mechanical preparation techniques, including polishing and dimple grinding, followed by a final thinning with ion milling.^20^ However, the so-called conventional preparation technique involves several drawbacks. For instance, the mechanical load during cutting or polishing can cause changes in the microstructure. The subsequent argon sputtering can lead to implantation, amorphization, and/or preferential sputtering in heterogeneous materials. A less invasive alternative for the preparation of plan-view specimens is, for instance, the wedge polishing method. It allows in some cases even an Ar-sputtering-free preparation.^21–26^

Another applicable method is the focused ion beam (FIB) lift-out technique, which is commonly used to fabricate cross-sectional TEM lamellae.^27–31^ This method allows preparing a small volume of the original material, thus leaving the remaining sample intact and available for further measurements.^32,33^ Moreover, FIB is suitable for a broad range of materials and allows the selection of the target area with a spatial accuracy up to the diameter of the ion beam.^8,33^ With its cutting, material deposition, and mechanical micromanipulation abilities, the FIB is the preferred instrument to prepare MEMS chips for *in situ* experiments with cross-sectional lamellae.^4,34,35^ However, in a wide variety of cases, the sole cross-sectional observation is insufficient for the reliable investigation of compositional or structural homogeneity of samples.^4,36^ Despite many advantages of the FIB previously highlighted, the plan-view FIB sample preparation is still not a common practice mainly due to demanding restrictions compared to the preparation of cross-sectional lamellae. Worthy efforts are directed toward novel approaches in plan-view sample preparation.^13,21,31^ Some methods rely on rotating the substrate 90° and then proceeding with already well-known cross-section lift-out. However, this technique is limited to near the surface region of the bulk material, and extended ion milling is necessary to achieve electron transparency. The latter together with the complex geometry can lead to changes in the material due to ion beam damage or re-deposition. Other techniques are based on milling channels around the predicted plan-view lamella and removing the material beneath it to detach the specimen from the bulk material. However, it is hard to observe whether there is still some material connecting the specimen with the bulk sample. The new preparation methods are expected to improve the success rate, efficiency, and quality of prepared specimens. In this regard, a number of studies have been engaged in solving obstacles faced during the plan-view preparation.^11,13,21,31,36–40^

It is noteworthy that an ion beam can cause temporary or permanent changes in the material. The main effects can be in the form of electrostatic charging, ionization damage, sputtering heating, and hydrocarbon contamination. Thus, for free surfaces with the structure of interest on it, ion beam illumination has to be avoided in all preparation steps. When an ion beam penetrates the target material, a series of interactions in the form of sputtering, amorphization, swelling, deposition, re-deposition, implantation and backscattering can occur.^6,30,41–45^

In this paper, we present a novel approach for sample preparation, which opens the world of *in situ* TEM heating experiments for a vast variety of fragile materials. The synergy of the mechanical wedge polishing technique with the advanced FIB lift-out procedure combines the advantages of both preparation methods and allows cutting a thin lamella in plan-view geometry and transfer it to a MEMS-based chip. This approach was testified using Si (001) with homogenously distributed Ge Stranski Krastanow (SK) islands grown by molecular beam epitaxy (MBE) as a model system.

## Materials and methods

**Figure 1:**
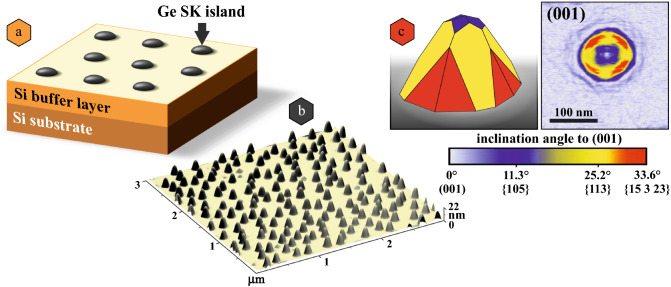
(**a**) a model of the investigated sample, (**b**) atomic force microscopy (AFM) height map (3 × 3 µm^2^) of self-assembled Ge islands formed upon deposition of 6 ML of Ge at 700°C on a Si (001) substrate, (**c**) the sketch of a single dome-shaped quantum dot as a surface angle image and the original AFM snapshot. The color coding represents the inclination of the facets with respect to the (001) surface. The facets {105}, {113}, and {15 3 23} are indicated in blue, yellow, and red color, respectively.

The model system, Ge SK islands, was grown in a SIVA-45 solid-source MBE chamber from Riber (France) on high-resistivity (> 5000 Ωcm) Si float-zone substrates. Prior to the growth, the samples were exposed to an RCA cleaning procedure^46^ and dipped in diluted hydrofluoric acid (HF 1%) before they were loaded into the MBE chamber. The H-passivated samples were degassed at a substrate temperature (*T*_S_) of 600°C for 25 min before the deposition of Si buffer layer of 135 nm thickness. The buffer was deposited at *T*_S_ ramped from 450 to 700°C and at a growth rate of 0.5 Å/s. The substrate cleaning, degassing, and the low initial *T*_S_ of a Si buffer layer ensure a clean interface between the Si (001) substrate and the epilayers.^47^ Subsequently, 6 monolayers ML (8.4 Å) of Ge were deposited at *T*_S_ = 700°C and a rate of 0.05 Å/s. Then, 1 nm of Si was deposited at 0.2 Å/s and a bias voltage of −2 kV at 60°C. This thin amorphous Si layer serves as a passivation layer to avoid oxidation of the underlying Ge epilayer.** Figure** **1** shows a schematic sample structure. At *T*_S_ = 700°C, either pyramidal islands or multi-faceted domes form.^48^ The growth conditions of the Ge layer were optimized^49^ to almost exclusively (> 96%) form islands of a dome shape with a very narrow size distribution with an average height of 20.2 ± 0.8 nm (see Figure 1b). In contrast to pyramidal islands that are bound to shallow {105} facets, 11.3° inclined to the Si(001) substate surface, domes are multi-faceted, exhibiting, in addition to {105} facets, also {113} (25.2°) and {15 3 23} (33.6°) ones. These facets are indicated in blue, yellow, and red colors in Figure 1c, where the color-coded local surface slope with respect to the (001) substrate surface is depicted.^50^ The chosen Si-based Ge island samples are an excellent model system for developing a new TEM sample preparation protocol for plan-view geometry. Firstly, Si becomes translucent below a thickness of 10 µm in visible light microscope (VLM) that can be used as a thickness indicator, as explained later. Secondly, Ge islands allow tracing the possible damage inflicted during polishing and the subsequent FIB-assisted installation.

For the wedge polishing, a MultiPrep (Allied High Tech Products Inc, US) instrument has been utilized, which uses diamond-lapping films (DLF, Allied High Tech Products Inc, US) as an abrasive. During processing, the sample was mounted on a Pyrex holder (TEM Wedge/FIB Fixture Body and Pyrex Inserts, Allied High Tech Products Inc, US).

A ZEISS Crossbeam 1540XB (ZEISS, Germany) scanning electron microscope (SEM) with FIB add-on was used to validate the wedge-polished plan-view samples and prepare the TEM lamellae on the MEMS chips. The angle between the SEM and FIB optical axes in this device is 54°, defining the tilt angles throughout our procedure. However, the preparation geometry can be easily adapted for other instruments. The lamella lift-out procedure and installation on a MEMS-based sample carrier was facilitated by the Kleindiek Nanotechnik MM3A-EM micromanipulator enhanced with the ROTIP-EM rotational axis plug-in. The FIB was operated at an acceleration voltage of 30 kV for sample lift-out and at 5 kV for the final thinning.

The *in situ* TEM heating experiment was carried out in a JEOL JEM-2200FS (JEOL, Japan) operated at an acceleration voltage of 200 kV. The TEM is equipped with an in-column Ω-filter and a TemCam-XF416 (TVIPS, Germany) CMOS-based camera. A state-of-the-art sample holder from DENSSolutions (Netherlands) was used, which utilizes MEMS heating chips.^51,52^ The procedure for the samples preparation presented in this work was applied to the Wildfire nano-chip (DENSSolutions, Netherlands). The heating element of the chip has a spiral configuration, and the membrane is heated by Joule effect. The heating holder has a 4-point probe connected to the nano-chip assuring accurate control during heating with high thermal stability.^5^

## Experimental

The first step of the advanced preparation of *in situ* TEM specimens is the fabrication of wedge-shaped samples in plan-view geometry. The primary goal is to mechanically polish wedges by removing material from the backside, leaving the surface intact and producing a large thin area. In this way, it is possible to cut several FIB lamellae from the edge, which is, ideally, electron transparent after mechanical polishing already. This allows serial fabrication of lamellae and reduces FIB milling time.^26^ The FIB is subsequently used to cut free and lift-out a suitable material slice, for mounting it to the MEMS chip and for the final thinning, polishing, and cleaning step. Backside polished material slices lead to an ideal cutting geometry, minimal re-deposition, and artifacts compared to a sole FIB plan-view preparation.

### Wedge-shaped sample preparation in plan-view geometry

For our wedge polishing, we followed mainly the methods described in Reference 23 and adapted them. Before the actual wedge preparation, several preliminary steps are necessary. First, the sample has to be cut to the appropriate size of 1.5 × 1.5 mm^2^ to fit with the inner diameter of the Cu-half grids used later. For this step, a high-precision wire saw (WELL Diamond Wire Saw 3400) was used. We utilized a wax (Hot Mounting wax, Allied High Tech Products Inc, US) and a heating stage (KA IKAMAG Combimag RCT Heated Magnetic Stirrer) at 100°C to fix the specimen on the saw sample holder. Generally, in order to avoid damaging the sample surface, it is important to press the sample only slightly into the wax when it is attached to a holder. The choice of the temperature is crucial and must be guided, keeping in mind the properties (e.g., thermal stability) of the investigated material.

**Figure 2:**
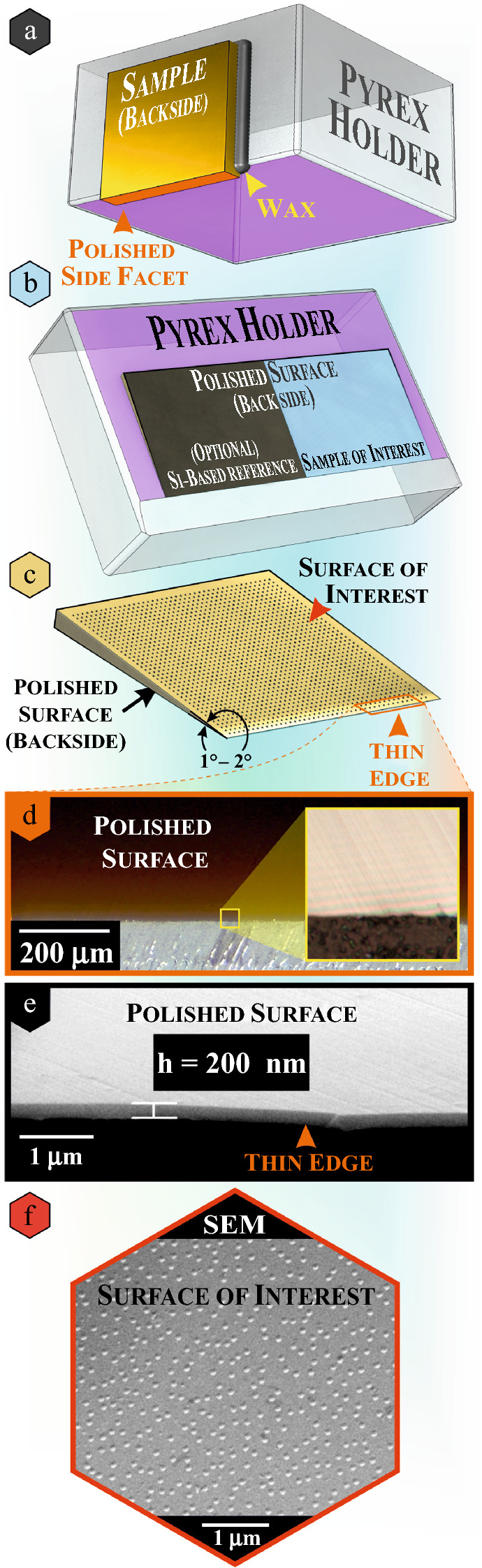
(**a**) the sample mounted on a Pyrex holder (side edge) for the primary side facet polishing step. (**b**) sample(s) on a Pyrex holder (bottom edge) for the wedge polishing step, another sample of interest can be installed together with a Si wafer reference (optional). (**c**) a model of a wedge-polished sample. (**d**) transmission VLM image of the wedge-polished Si sample thin edge depicting a color gradient and optical fringes (inset), which can be used for sample thickness determination. (**e**) the SEM image of the sample edge with a thickness below 1 µm, which was achieved via wedge polishing. (**f**) SEM image of the sample surface with Ge islands.

Second, the saw cut surfaces were polished using the MultiPrep device. The surface of interest was directly attached to the side edge of the Pyrex in such a way that the facet to be polished protrudes slightly (< 1 mm) over the holder (**Figure** **2**a). All four saw cut side facets were polished in this way using abrasive papers and DLF with a grain size of 0.1 µm during the final step. This procedure is crucial for the following steps since scratches in the side facet introduced during sawing are potential starting points for specimen cracking during the wedge polishing (thin edge in Figure 2b).

After these primary preparations, the sample was attached with wax to the bottom edge of the Pyrex holder of the MultiPrep. Pyrex holders are consumable parts, and during the wedge polishing, a shallow chamfer can be produced on it. Thus, it is essential to planarize the Pyrex holder before each new wedge preparation by polishing it with DLF (grain size of 1 µm or smaller), ensuring a flat and scratch-free surface. A small amount of wax was melted on the Pyrex, and the sample was placed with the facet that will become a thin edge as close as possible to the front edge of the Pyrex (Figure 2b). Again only slight pressure was applied to ensure a uniform thin wax layer between the sample and the holder and to avoid damaging the surface of interest. The initial thickness of the test Si-based sample was 550 µm. The steps applied during wedge polishing are summarized in **Table** **I**, including the choice of diamond-lapping films, polishing duration, the quantity of removed material, and the disk rotation speed (revolutions per minute—RPM). We used a denatured alcohol-based lubricant and cooling agent with low viscosity (commercially available BlueLube Polishing Lubricant by Allied High Tech, US). It accelerates the polishing procedure (accelerates material removal during polishing) and helps in maintaining the durability of the polishing cloth. Additionally, deionized water (DI H_2_O) was used before, during, and after polishing for wetting, diluting, and cleaning the DLF and lubricant. The removed material leaves stains and traces on DLF, which must be rinsed off with DI H_2_O/ethanol and a rubber squeegee. Generally, during the entire procedure, a load of 500 g was applied; however, as the sample becomes thinner, the load can be set to lower values to avoid damages to the polished surface or edges.

**Table I:** Summarized steps of the wedge polishing procedure.

DLF [μm]	Approx. time [min]	Final thickness [μm]	Rotation direction (Si-based samples)	Speed [RPM]
6	15	350	Toward the thin edge	50
3	15	250	"	50
1	10	200–150	"	50
1^a^	20	50	"	30–35
0.5^a^	10	50–20	"	30–35
0.1^a^	10	20–1	"	30–35
OPS^a, b^		< 1	Toward the thick edge	30–35

^a^The inclination angle α is set to the value of 1°–2°

^b^Optional step for polishing the specimen down to electron transparency

An inclination angle α was introduced when the thickness of the sample reached approximately 250–200 µm. Different values have been tested for α. An inclination of 1–2° was found to yield the most mechanically stable, high-quality thin edges for Si-based specimens (Figure 2c). The inclination was introduced to the polishing procedure using DLF with a grain size of 1 µm after the scratches originating from the previous DLF (3 µm) had been removed.

Adequate exchange of DLF with smaller particle sizes is directly correlated to the decrease of specimen thickness during preparation. Faulty DLF change can lead to cracks, deep scratches, and amorphization.^20^ Before changing to a finer DLF, thorough cleaning of the sample is very important. The abrasive particles from the previous steps have to be removed without destroying the wax connection of the sample and the Pyrex. Between the polishing steps with different films, the sample was rinsed with ethanol and DI water. Additionally, slight mechanical cleaning with a cotton swab and micro-organic soap (Allied High Tech, US) can be used to remove abrasives stuck in the wax. Wax residues can be removed easily with acetone, but in order to maintain the bond between the sample and the holder, no acetone was used for cleaning during polishing.

One of the main challenges of the wedge polishing technique is the determination of the thickness of the sample. The sample’s thickness was continuously tracked by means of the translucency of Si, indicated by the color getting brighter. The sample color was monitored with a VLM. The red color appears when the Si sample thickness is 5–10 μm, light yellow at ≈ 1 μm, and Newton's rings become visible when the sample is less than 1 μm thick (see Figure 2d).^53–55^ In this regard, the chosen Si-based samples are an excellent model system for developing a new preparation protocol for plan-view geometry due to this inherent thickness indicator. For other materials, a piece of Si can be installed side by side during polishing and used as a reference for a general thickness determination (Figure 2b). We have already successfully tested this approach with bulk Ge-based samples. These specimens, in contrast to the Si-based ones, stay opaque at thicknesses below 1 µm.

In this way, the samples could be wedge-polished quite fast to a thickness below 1 μm. Here, a final optional polishing step with the oxide polishing suspension (OPS) (see Table 1) can be applied to fabricate specimens directly suitable for *ex situ* TEM investigation (≈ 200 nm thick for Si) without further thinning (Figure 2e). However, *in situ* TEM experiments require FIB-assisted preparation of specimens on MEMS-based sample carriers. For this procedure, the wedge-polished sample must be mechanically stable for installation on the MEMS chip. Thus, as soon as the wedge became thinner than ≈ 2 µm, the polishing was terminated and we have proceeded with the next preparation step.

Achieving the desired final thickness, the wedge-polished sample can be detached from the Pyrex holder utilizing an acetone bath. The Pyrex holder with the specimen was dipped in a beaker filled with acetone. When the wax dissolved, the sample was detached from the Pyrex and sank down to a paper tissue placed on the bottom. For cleaning, we exchanged the acetone three times by pipetting and then switched to ethanol and finally DI water. Both were replaced several times to end up with a clean solvent. Additionally, baths with micro-organic soap and GP cleaning solution (Allied High Tech) can be used to remove residues of wax if present. Once cleaned, the wedge sample together with the anti-static paper was taken out of the beaker and dried.

We have used copper rings cut in half as sample carriers for a FIB-assisted installation on the MEMS chip. The best approach here is to place a small amount of epoxy resin (M-Bond 610 from Micro-Measurements) on a copper half-grid. By moving only the ring with tweezers, one can glue the ring onto the fragile, thicker edge of the wedge. This allows mounting the wedge to the copper grid without handling the fragile wedge directly with tweezers. After resin hardening, the wedge can be handled utilizing copper ring support, which allows additional cleaning.

The quality of the prepared plan-view wedge-shaped sample was first validated using SEM, where the thickness of the thinned edge was determined. Thus, the unpolished surface was inspected for any specks of dirt, residuals of cleaning solutions, dust particles, mechanical damages such as cracks and scratches, etc. Figure 2f shows that the applied procedure leaves a clean surface with well-distinguishable SK islands that correlate well with the data presented in Figure 1.

### Preparation of a MEMS-based heating chip

**Figure 3:**
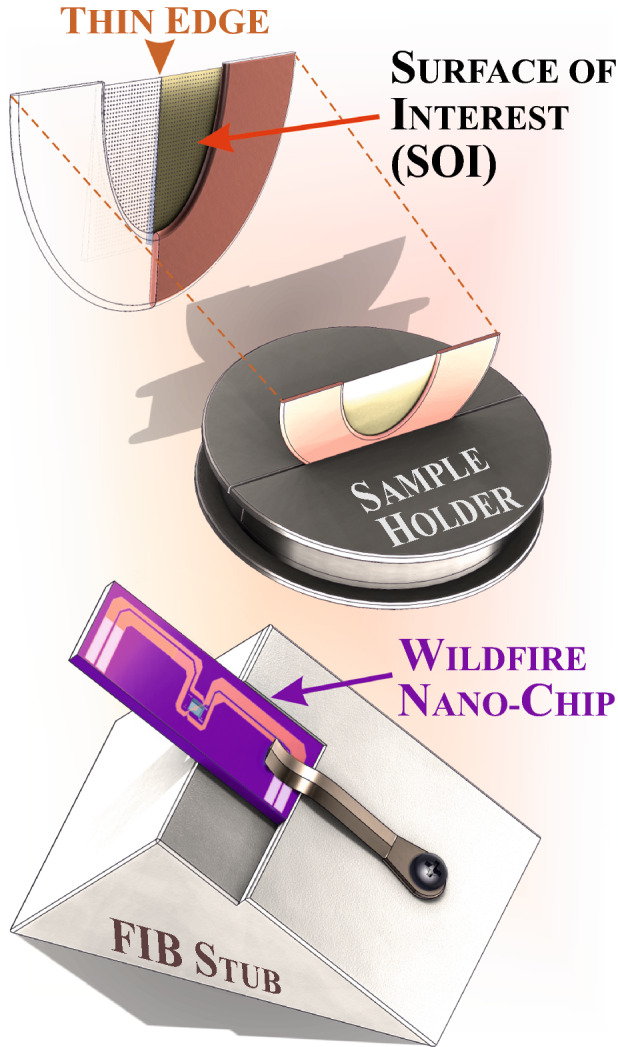
The tools for the FIB-assisted sample installation on a MEMS-based heating chip. TEM copper half-grid with the glued sample (top), the half-grid installed in a custom-designed sample holder^56^ (middle), a Wildfire nano-chip (JEOL-compatible) fixed on a special FIB stub (bottom).

While developing this approach, we have focused on preserving the physicochemical properties of the original surface (SK islands). Thus, the main intention, which governs the new alignment geometry, was to avoid detrimental FIB illumination of the surface of interest. The wedge-shaped sample was mounted parallel with the surface of interest directly attached to the copper TEM half-grid (see** Figure** **3** top) used as a sample carrier. The half-grid was installed in a custom-designed sample holder for SEM,^56^ which assures the sample surface being perpendicular to the SEM stage (Figure 3 middle). This sample orientation allows FIB cutting and thinning from the backside of the sample keeping the surface of interest ‘in a shadow’ of the ion beam illumination. The MEMS nano-chip, in its turn, was placed on a special in-house FIB stub in a way shown in Figure 3 (bottom). This peculiar alignment was motivated by methodological reasons and noticeably facilitated the installation process.

The FIB-assisted installation of the sample on the MEMS chip can be divided into one optional and three main steps:

1. preliminary thinning (optional step, additional to wedge polishing);
2. lamella lift-out;
3. *in situ* transfer to the MEMS chip;
4. final specimen thinning.

#### Preliminary thinning (optional)

If the prepared wedge-shaped sample is thicker than 2 μm, it is necessary to apply the additional thinning with FIB before proceeding with the lamella lift-out. First, before switching on the FIB gun the sample must be aligned in a way its surface of interest directed toward the FIB gun and then tilted β + 2°, where β is the angle between electron and ion guns (in our case 54°). The sample should be over tilted to avoid ion beam illumination of the surface with the SK islands resembling the approach suggested by Gries et al.^57^ It is noteworthy that no capping layer was deposited on the sample edge since this could affect the surface properties through precursor diffusion and contamination. However, in most cases, the capping layer deposition is vital for polycrystalline materials to reduce the curtaining effect. After alignment, the so-called C-bar FIB back-milling procedure^40^ could be performed with the ion beam operated at 30 kV and 5 nA.

**Figure 4:**
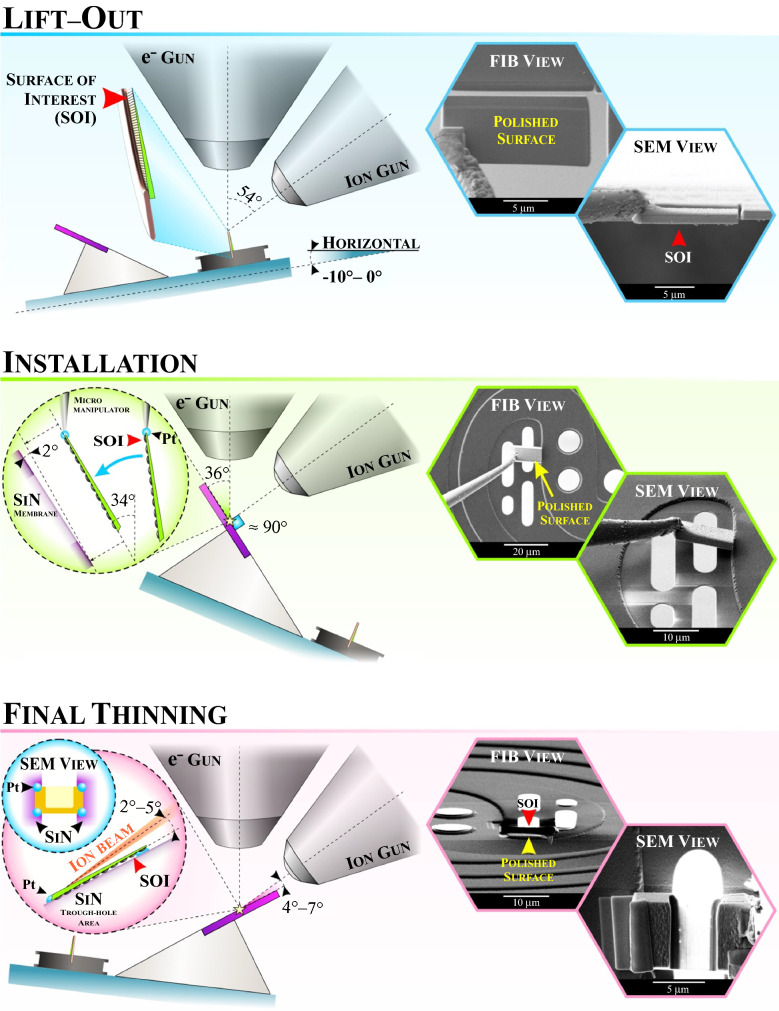
The sample on-chip preparation guideline. General alignment geometry for the main steps and the correspondent FIB and SEM images.

Suggested alignment geometries for the main three steps of the sample on MEMS chip installation accompanied with correspondent FIB and SEM images are shown in** Figure** **4**. Obviously, all mentioned angles are specific for the used equipment, but can be easily adapted following the general presented approach.

#### Lamella lift-out

If a wedge-polished sample is thin enough (bellow 2 μm), a plan-view lamella can be directly cut out from the thin edge. The mutual alignment of the sample holder and the nano-chip in the chamber is schematically depicted in the corresponding section in Figure 4. Note that we are constantly protecting the surface with the islands and keeping it opposite in regard to ion gun. The SEM stage at this moment is suggested to be aligned in the -10°–0° range to the horizontal. Utilizing a counter-clockwise stage tilting could facilitate the following sample-to-chip transfer procedure (minimizing the micromanipulator rotation). After selecting the desired region, the thin edge of the sample must be precut. Using the cut gap, the micromanipulator is brought into contact with the top left corner of the predicted lamella and attached to it with ion-stimulated Pt deposition (see also the lift-out section in Figure 4). Then the lamella can be cut free, operating ion beam at 30 kV 500 pA conditions.

#### *In situ* installation on the MEMS chip

First, the stage should be safely tilted clockwise, while the chip surface should be nearly perpendicular to the ion beam. The rotating manipulator drastically eases the transfer of the lamella allowing adjustment of the angles mismatch between the surfaces of the chip and lamella (please see the installation section in Figure 4). To facilitate the last step of the procedure (the final thinning), the lamella must be intentionally attached to the chip over the through-hole area at a slight angle of approximately 2°. Note that the surface of interest is faced down to the SiN membrane (see the inset); consequently, it is not affected by an ion beam.

#### Final thinning

For the final thinning step, the SEM stage was rotated 180° and tilted in a way shown in Figure 4. This sequence allows to remove material from the backside, leaving the surface of interest protected from the damaging effect of ion milling. The final thinning down to the thickness of < 100 nm was performed gradually reducing the ion beam accelerating voltage and current from 30 kV/500 pA to 5 kV/50 pA at 2° and 5°, respectively (see the inset), applying the adapted C-bar FIB back-milling approach.^40^ Thinning to electron transparency was traced in real-time by SEM imaging. The electron transparent area of the lamella during thinning appears brighter in the SEM image (Figure 4, the final thinning section). In the frame of the current investigation, the ion milling has been interrupted when electron transparency at the acceleration voltage of 3 kV was achieved (Figure 4, the final thinning section, SEM view). After accomplishing the sample installation, the quality of the prepared sample was immediately validated via TEM.

## Results and discussion

**Figure 5:**
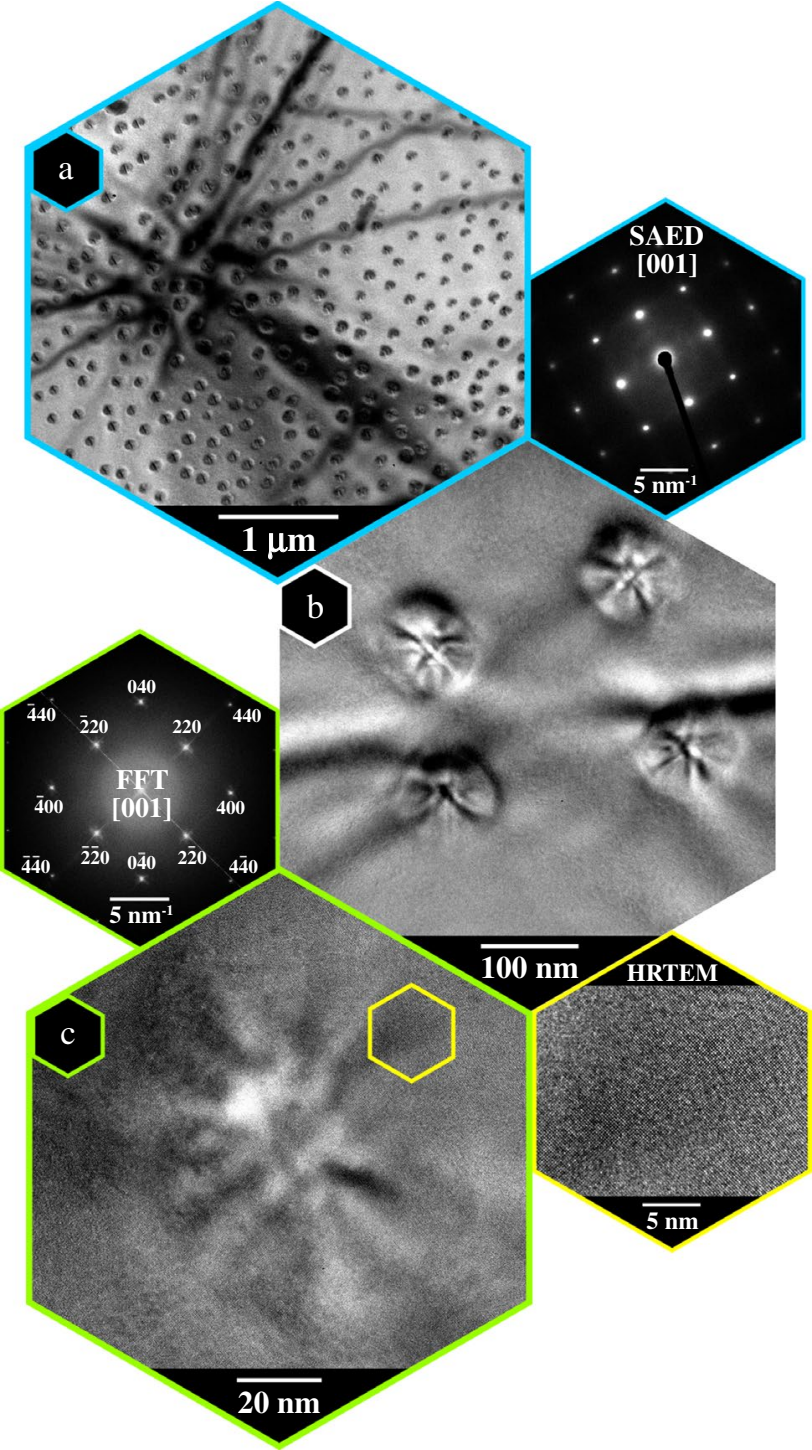
The plan-view sample, installed on a MEMS-based chip and pre-checked via TEM at room temperature. (**a**) TEM image of the sample aligned approximately along [001] and the corresponding SAED pattern, (**b**) enhanced imaging of a Ge island area on the Si substrate, and (**c**) HRTEM snapshot of one Ge island together with the correspondent FFT pattern and the magnified image of the area highlighted with a yellow hexagon.

The quality of the specimens has been evaluated first at room temperature via light and electron microscopy. SEM and TEM images of SK islands from the plan-view wedge-shaped specimen and the sample prepared on the MEMS chip are comparable (see** Figures** **2**f and **5**a). Likewise, the surface is clean and free of dirt or cleaning solution residuals, as well as unwanted Pt deposition. The general bright field (BF) TEM image of the prepared plan-view lamella mounted on the MEMS chip is presented in Figure 5a. The lattice constant mismatch between pure Ge and Si is around 4%. Thus, Ge on Si builds up a strained system. This leads to the SK growth mode and the formation of the Ge islands. In thin plan-view specimens, backside thinned from the Si substrate, Ge can expand and release some of the strain leading to a spherical bending of the thinned area. This can be seen in the zone axis pattern (ZAP) – a contrast formed by the bending contours in the BF image in Figure 5a. The specimen was aligned parallel to the [001] zone axis, which was determined by selective aperture electron diffraction (SAED) (Figure 5a, inset). At the crossing of the bending contour in the ZAP, the specimen orientation is perfectly achieved. Being aligned along a zone axis, the islands show a characteristic contrast that results from a combination of strain and thickness effects and is determined by the high-order symmetry of perfect SK islands (Figure 5b).^58,59^ This symmetric contrast is an indicator for a coherent island without abrupt crystallographic changes caused by dislocations or stacking faults, which would lead to relaxed Ge on top of Si exhibiting a Morié pattern contrast.^60^ One example is depicted in Figure 5c together with the correspondent power spectrum calculated by fast fourier transformation (FFT). No distortions are visible in the TEM contrast, indicating high crystal quality and a defect-free island. Additionally, minor traces of amorphous material are visible in the FFT pattern, indicating a negligible amorphization of the specimen during wedge polishing and FIB-assisted installation. Also, no other diffraction reflexes, e.g., from unwanted Pt deposition, could be found. The thickness of the underlying Si material slice was estimated to be approximately 100 nm. This thickness assures a stable enough supporting Si membrane for the heating experiment. Overall, these measurements prove that a transfer of sensitive, only a few nm high nanostructures like the Ge islands, from a bulk substrate to MEMS heating chips in plan-view geometry can be achieved without damaging the structure of interest. The next step was testing the performance of the prepared specimen in a heating experiment.

While tilting the sample away from the zone axis, the contrast of the coherent islands losses its symmetry. The dynamical diffraction fringes of the applied Bragg diffraction condition form a line pattern containing bright and dark lines. To highlight the inherent strain of the SK islands during the *in situ* heating, the two-beam condition (TBC), at which the −2−20 diffraction reflex is excited has been applied. Bright-field images recorded at this condition show the so-called butterfly contrast at the positions of the islands.^59,61^ The dark “lines” present in the center of the Ge islands are caused by the missing intensity in the direct beam, which is diffracted into the −2−20 reflex. Such Bragg diffraction contrast lines are highly sensitive to structural crystal properties and are used, for example, for Burgers vector determination with large angle convergent electron beam diffraction or the contour line method.^62^ By this contrast, the introduction of strain relaxing defects like dislocations or stacking faults can be visualized directly by a splitting of the above-mentioned “lines”. Even if the process of dislocation reactions is too fast to be traced directly by our camera system, the changes at the SK islands and, in particular, the appearance of dislocation and stacking faults during an *in situ* heating experiment can be well monitored applying this imaging mode.

**Figure 6:**
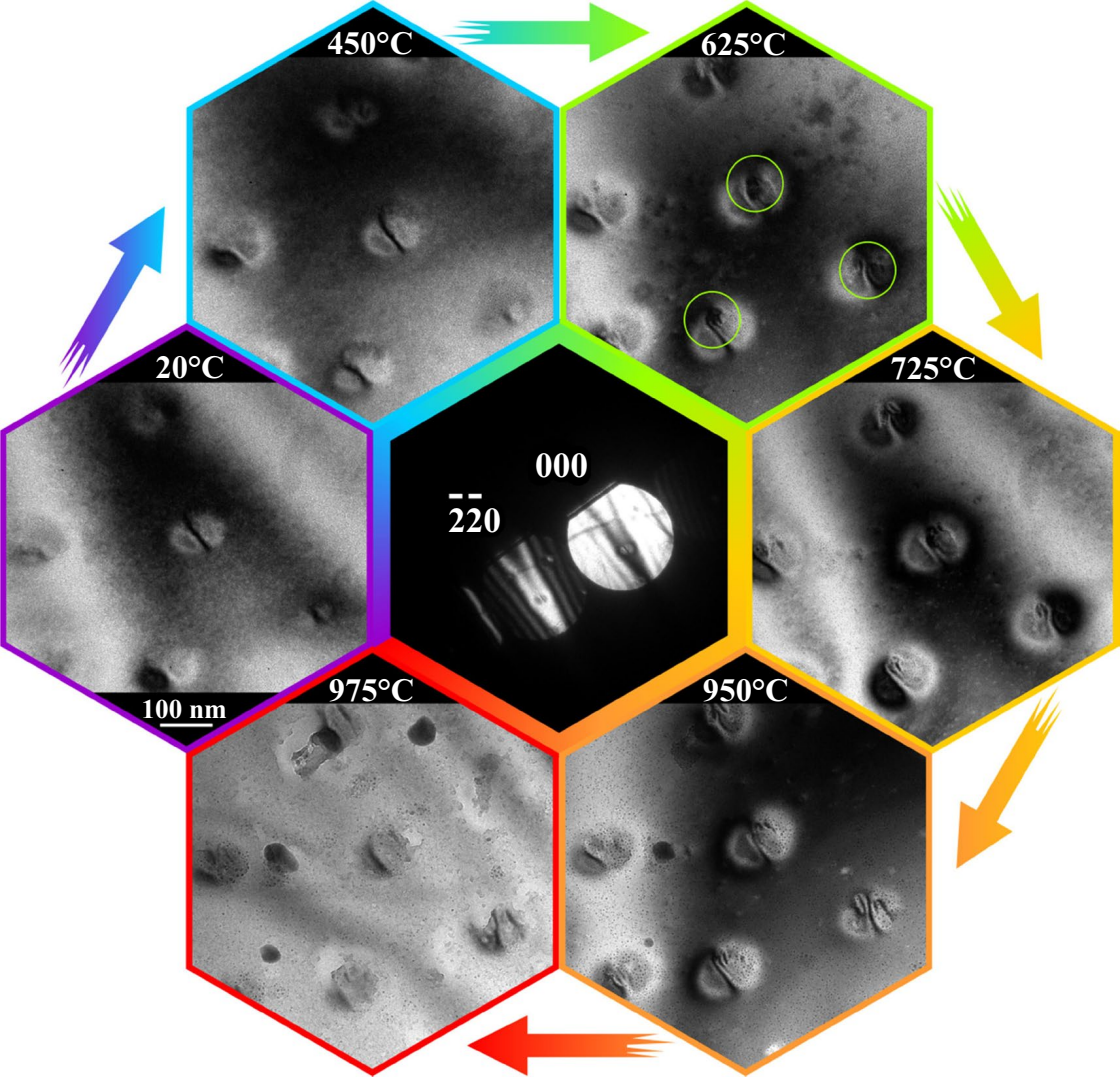
Selected TEM images of the sample at different temperatures taken under two-beam condition (inset in the center) recorded during a continuous *in situ* heating experiment. The formation of dislocations within the Ge islands at 625°C is highlighted with circles. Full animated video can be found in the supplementary materials.

The used model system exhibits one challenge that is generally inherent to strained heterostructures. Strain variations induced by different thermal expansion coefficients led to bending of the thinned area during heating and is inducing orientation changes. Two possible options are available for compensation. First, during continuous heating, a larger area can be monitored (like in Figure 5a), and by following the ZAP during heating, similar orientated islands can be compared at different temperatures. Second, if one wants to follow precisely the same group of islands during heating, one can apply the step-wise heating approach, which we have selected. After each heating step, the sample orientation is realigned. Thus one group of islands can be traced for different temperatures at exactly the same diffraction condition. Observations of the behavior of the Ge islands on Si substrate during the *in situ* heating experiment are demonstrated and supported by a TEM image sequence of the same area of the sample at different temperatures varied in the 50–1000°C range with a step of 25 K (**Figure** **6**).

While heating, the strain in the islands increases because the linear thermal expansion coefficient of Ge (*α*_Ge_ ~ 6·10^–6^ 1/K) is larger compared to that of Si (*α*_Si_ ~ 3·10^–6^ 1/K at 20°C). Thus, one can see additional bending and a slight change in the TBC strain contrast (Figure 6). The strain level can eventually reach the threshold for dislocation formation, which in the case of partial dislocations leads to stacking faults appearance. Indeed, one can see the formation of such defects at around 625°C, indicated by the emergence of curved, split contrast lines within the Ge islands (highlighted with circles in Figure 6), which is a well-known sign of the dislocation presence.^63^ A rough estimation applying the difference in the thermal expansion coefficient (Δ*α* ~ 3·10^–6^ 1/K) and the temperature distinction of 600 K leads to an ≈ 0.18 nm mismatch in thermal expansion for a 100 nm large Ge island and the Si substrate. This value fits well the Burgers vector length of a partial dislocation (*b*_Ge, partial_ = *a*/6 < 112 > , *b*_Ge, partial_ = 0.23 nm) in the Ge diamond structure system, which is a strong indication that at this temperature a single stacking fault appears. After heating and cooling down, the defects remain in the islands proving an overall, permanent structural change.

At higher temperatures (725°C), one can notice (Figure 6) the undesirable effect caused by the Pt used for lamella fixation on MEMS chip. Elevated temperature stimulates Pt diffusion over the free surface. Consequently, the surface of interest is contaminated by small Pt islands. If other more refractory deposition materials like tungsten will show similar effects and which metal precursor is better suitable for different material systems is still to be tested. The melting point of Ge is at 938.2°C, and the image taken at 975°C shows that the primary Ge island structure is changed due to the melting process. Generally, our test shows that SK island can be prepared for *in situ* TEM heating with the developed approach, which will allow investigating relaxation and defects healing in these structures on the fly. The method should be applicable for similar semiconductor and thin layer systems, where temperature-induced effects like decomposition can be monitored.^4,64^

## Conclusion and outlook

*In situ* plan-view TEM investigations of thin-layered systems deliver a good overview of the sample's properties and allow monitoring of surface-related, temperature-induced effects in real time. It has been shown that the suggested combination of wedge polishing and FIB-assisted specimen transfer to MEMS chips is a well-suited approach for the preparation of plan-view specimens. We have identified various advantages of the presented method. The mechanical pre-thinning from the backside by wedge polishing minimizes FIB processing time and facilitates the FIB lift-out routine. In this way, detrimental Ga ion illumination is reduced, while re-deposition of the sputtered material on the sample’s surface can be avoided. This allows the transfer of an unaffected surface to the chip while mounting, thinning, and final cleaning can be done with FIB in a way similar to the cross-section lamellae preparation routine. This is achieved by orientating the surface of interest toward the chip. Additionally, the thinned wedge-shaped specimen is well suited for cutting multiple lamellae from the same specimen and thus facilitates *in situ* attaching a series of lamellae to MEMS chips giving a base to a thorough systematic study of materials properties.

We have tested the developed approach using epitaxial Ge islands on crystalline Si to understand their strain relaxation mechanisms upon thermal exposure. Major strain relaxation in the islands occurs through the formation of stacking faults induced by different thermal expansions coefficients of the involved materials at 625°C. Apart from this prototype system, *in* *situ* TEM annealing experiments are approachable for a broad class of material systems due to the flexibility of the combined wedge polishing and FIB methods presented in this work.

## Supplementary Information

Below is the link to the electronic supplementary material.

Supplementary file1 (MP4 8754 kb).

## Data Availability

The data that support the findings of this study are available from the corresponding author upon reasonable request.
